# Supplementary material for: Unconstrained coevolution of bacterial size and the latent period of plastic phage
Source: PLoS One. 2022 May 26;17(5):e0268596. doi: 10.1371/journal.pone.0268596 (PMC9135238; doi:10.1371/journal.pone.0268596)
Supplement: S1 File — (PDF) [file pone.0268596.s001.pdf]

Supplementary Information

Host-use minimization strategy when only virus evolves

It is possible to calculate analytically the optimal latent period of a virus that evolves trying to minimize the requirement of a (nonevolving) host. In other words, the latent period that minimizes the density of hosts needed for the survival of the virus phenotype. This would be equivalent to finding the virus with the smallest "R^*", using the jargon of resource competition theory [1].

Assuming that the dynamics of the system follow Eqs.(1)-(4), setting the equations to zero provides the condition for the stationary state. For example, from setting the r.h.s. of Eqs.(1) and (3) to zero, we obtain the expression:

$$C^* = \left[\mu^* + \left(\frac{k}{\alpha_V} - 1\right) w + \frac{k}{\alpha_V} m\right] \left[\frac{k^2 \left(B e^{-w_{out} L} - 1\right)}{\alpha_V} + \alpha\right]^{-1}, \tag{S1}$$

where the asterisk represents "value at the stationary state". Because the host does not evolve, the value of the latent period that minimizes the expression given by Eq.(S1) needs to fulfill:

$$\frac{dC}{dL} = 0. \tag{S2}$$

Given the expression for C^* above, such a condition is fulfilled if the derivative of the denominator in Eq.(S1) is zero, which ultimately leads to the condition:

$$\frac{dB}{dL} = w_{out}\ B. \tag{S3}$$

Since the burst size is linked to the latent period through the tradeoff function Eq.(9), the condition above translates into:

$$L_{nocoev} = \frac{1}{w_{out}} + E. \tag{S4}$$

Thus, Eq.(S4) describes the evolutionarily stable strategy (ESS) for a virus for which evolution aims to minimize the host density needed for survival, in the presence of a host that does not evolve. This expression is identical to the ones obtained in the past for other versions of the delay model when calculating the optimal latent period of the virus [2-4].

Supplementary Figures

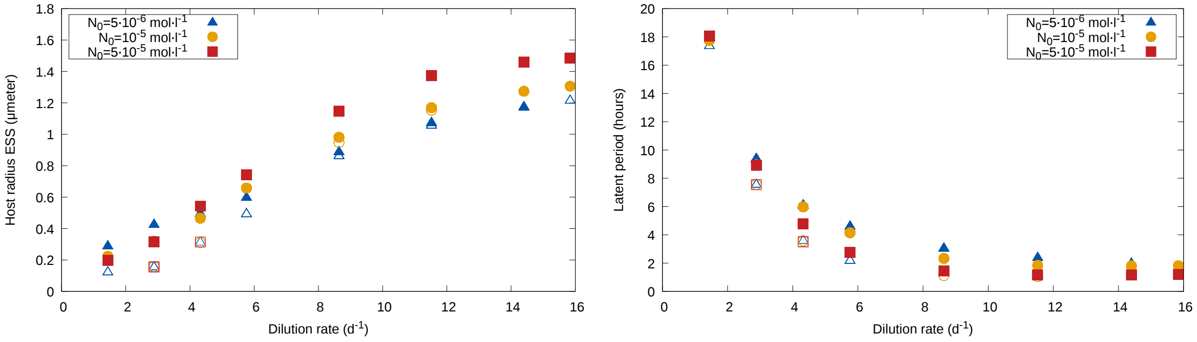

Figure S1: Dependence of the evolving traits on the dilution rate, w. The emergent r_{ESS} value (left) increases with w and input concentration, N_0, whereas the latent period decreases (right). Warmer colors indicate increased input concentrations.

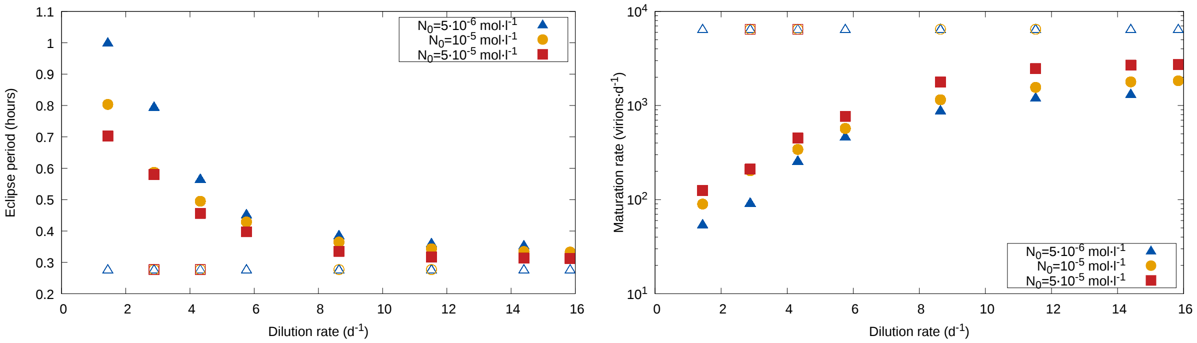

Figure S2: Eclipse period (left) and maturation rate (right) as a function of the dilution rate. Both are constant for the nonplastic case, but depend on growth rate in a negative and positive way, respectively, in the plastic case.

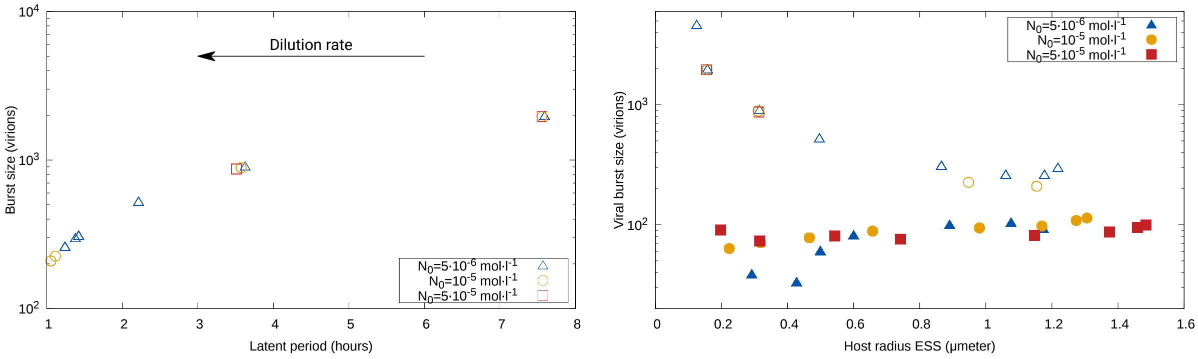

Figure S3: Left: Burst size as a function of the emerging latent period for the nonplastic case, which shows a positive correlation as expected from using Eq.(9) with fixed E and M, and the nonplastic L_{ESS} from Fig. 3, left panel. Right: Average across replicates of emerging burst size as a function of host radius; the plastic case shows a mild positive correlation, whereas the nonplastic version shows a marked negative correlation.

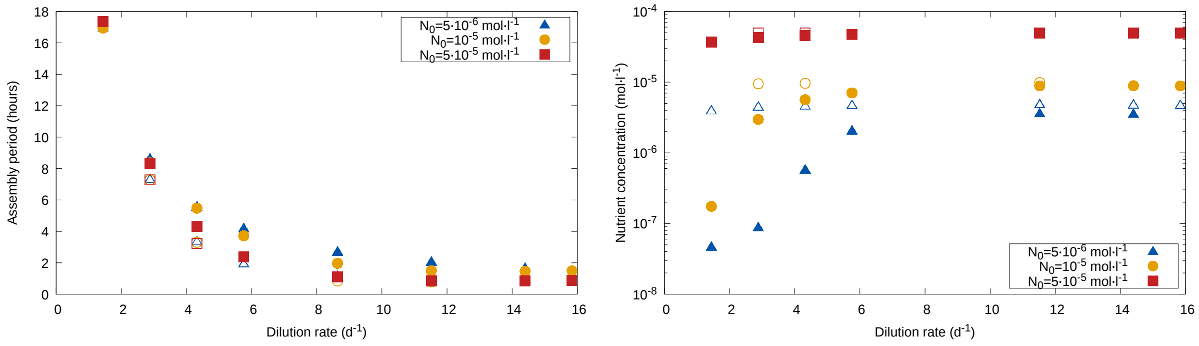

Figure S4: Left: Assembly period (time between end of eclipse period and lysis, $L - E$) as a function of dilution rate. Right: Average across replicates of nutrient concentration as a function of w; the plastic case shows lower N for low dilution rates, but both converge to the input concentration N_0 as w increases.

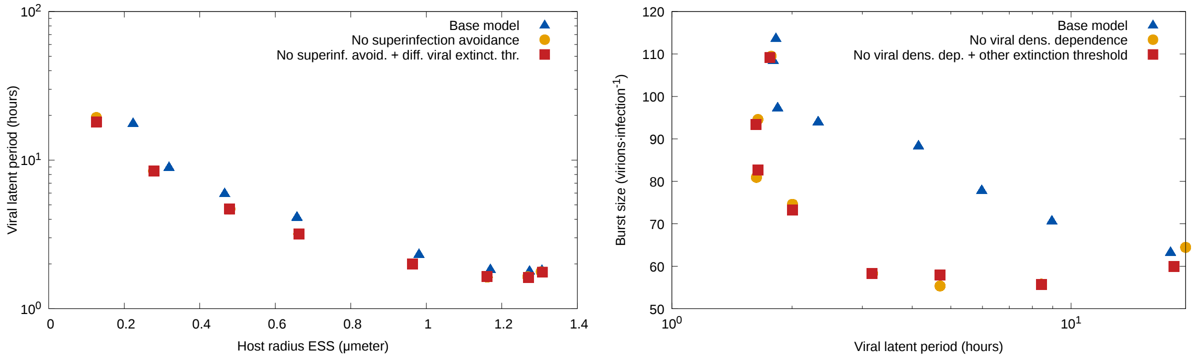

Figure S5: Emerging traits for other versions of the plastic model (see main text). Left: Host radius as a function of viral latent period, for which the base model (i.e. main model described in Methods) shows results that are barely distinguishable from the case with no superinfection avoidance (i.e. with $\alpha_V = 0$) and from the case with a threshold viral for extinction that depends only on free virus availability. Right: Burst size as a function of the latent period, which shows a qualitatively similar decreasing trend for all three versions, but the base model produces longer infections with larger burst sizes.

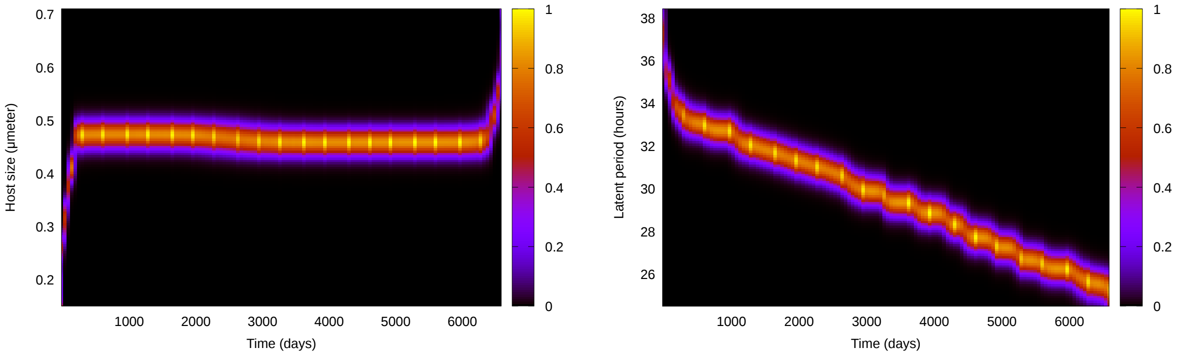

Figure S6: Evolutionary paths to system collapse for the plastic case with $w = 10$, $N_0 = 5 \cdot 10^{-5}$. Left: Host radius, r. Right: Viral latent period, L.

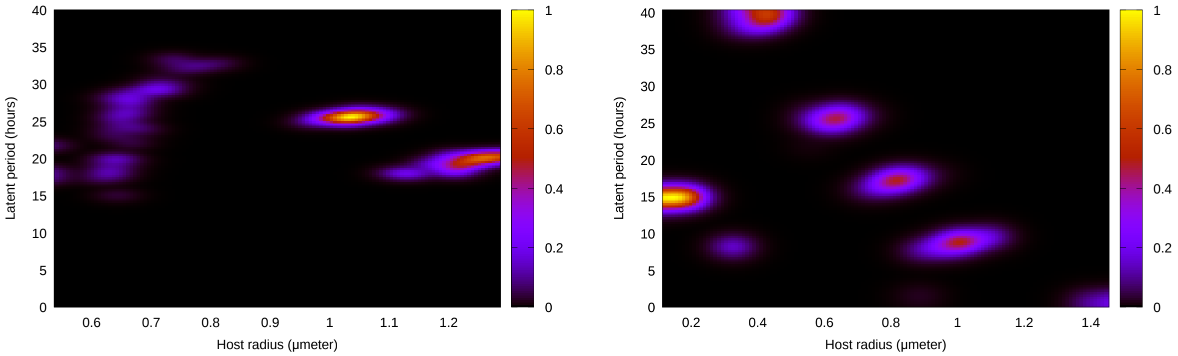

Figure S7: Representative examples of (normalized) density plots for the host radius and viral latent period observed immediately before evolutionary collapse of the system occurred for the plastic ($N_0 = 5 \cdot 10^{-5}$ $mol \cdot l^{-1}$, left) and the nonplastic ($N_0 = 10^{-5}$ $mol \cdot l^{-1}$, right) case. See Fig. S6 for examples of evolutionary path to extinction for the plastic case. As in Fig. 2, dilution rate increases from left to right.

References

[1] Tilman D. Resource Competition and Community Structure. 1st ed. Princeton University Press; 1982.

[2] Choua M, Bonachela JA. Ecological and Evolutionary Consequences of Viral Plasticity. Am Nat. 2019;193:346–358.

[3] Bonachela JA, Levin SA. Evolutionary Comparison Between Viral Lysis Rate and Latent Period. J Theor Biol. 2014;345:32–42.

[4] Shao Y, Wang IN. Bacteriophage Adsorption Rate and Optimal Lysis Time. Genetics. 2008;180:471–482.
